# Protein-Targeting Drug Discovery

**DOI:** 10.3390/biom13111591

**Published:** 2023-10-29

**Authors:** Caterina Vicidomini, Giovanni N. Roviello

**Affiliations:** Institute of Biostructures and Bioimaging, Italian National Council for Research (IBB-CNR), Area di Ricerca Site and Headquarters, Via Pietro Castellino 111, 80131 Naples, Italy

Protein-driven biological processes play a fundamental role in biomedicine because they are related to pathologies of enormous social impact, such as cancer, neuropathies, and viral diseases, including the one at the origin of the recent COVID-19 pandemic [1]. Knowing the structure of the protein target is the first step in the rational design of inhibitors or compounds with ad hoc modulating activity of the target protein function, to be used as therapeutics. One of the several protein-targeting strategies utilized in the field of neurotherapeutic drug discovery consists of the selective inhibition of gamma-aminobutyric acid (GABA) transporter type 1 (GAT-1, Figure 1a), which leads to increased levels of the inhibitory neurotransmitter GABA within synapses [2].

The study of the structure of the human GAT-1 in complex with the antiepileptic drug tiagabine (**1**, Figure 1b), and particularly of the main binding site for **1,** is fundamental for the rational design of new neurodrugs acting as inhibitors of GABA transport [3,4]. Targeting proteins is also a winning strategy in the antimicrobial drug discovery process. For example, inhibiting the enzyme nicotinamidase of *Plasmodium falciparum* could lead to potential antimalarial effects and remarkably, since humans lack this enzyme, nicotinamidase inhibitors are expected to be safe drugs [5]. Ribonucleotide reductase (RR), a multi-subunit enzyme that catalyzes the formation of deoxyribonucleoside diphosphates from their ribonucleoside analogs, is an attractive therapeutic target for a number of proliferative pathologies, including cancer, a pathology against which numerous synthetic molecules are being tested [6,7,8,9], since the expression levels of this enzyme are typically high during cell replication [10,11,12]. There are different inhibitors of human RR that are potential anticancer drugs including the nucleoside analog inhibitors of the large catalytic subunit RRM1, such as clofarabine (**2**, Figure 1b) and gemcitabine (**3**), as well as the inhibitors of the free radical housing small subunit RRM2, such as hydroxyurea (**4**) and triapine (**5**) [13,14,15]. Among the post-translational modifications of proteins, ADP-ribosylation of proteins is a post-translational modification involved in cancer and thus, enzymes involved in monoADP-ribosylation/polyADP-ribosylation cycling are drug targets for cancer therapy [16]. The search for efficacious therapies for COVID-19 motivated the scientific community to investigate the interaction of natural compounds, such as the stilbene polyphenols resveratrol (**6**) and polydatin (**7**, Figure 2), with the SARS-CoV-2 spike protein and its main receptor ACE2 (Figure 2a,b) [17,18,19,20].

Famously, the spike protein is essential for SARS-CoV-2 entry into human cells, while ACE2, the angiotensin-converting enzyme found on the surface of respiratory epithelial cells and several other host cell types, is the main receptor for the spike protein. Thus, therapeutics including anti-COVID-19 drugs can be inhibitors or modulators of protein–protein recognition. Protein binding is not only used to block a pathologic process at the molecular level, but can also be used for drug delivery applications; in this regard, serum albumin binding of synthetic molecules or metal complexes [21] was recently investigated to improve the transport of potential drugs in the human body [22,23,24,25]. Finally, tumor-associated macrophages are known to exert different pro-tumoral functions, promoting not only proliferation, invasion, and angiogenesis, but also immune tolerance and therapeutic resistance. The proteins expressed on tumor-associated macrophages are considered attractive targets for anticancer therapy in strategies aimed at either inhibiting the pro-tumoral functions of these cells or reducing their levels [26,27,28]. In conclusion, protein-driven biological processes are highly connected with disease and inhibiting or modulating protein functions with specific pathological implications can be an effective weapon in the search for new therapies for a diversity of pathologies that affect humans including those with the highest social impact such as COVID-19, cancer, infectious diseases, and neuropathies.

## Figures and Tables

**Figure 1:**
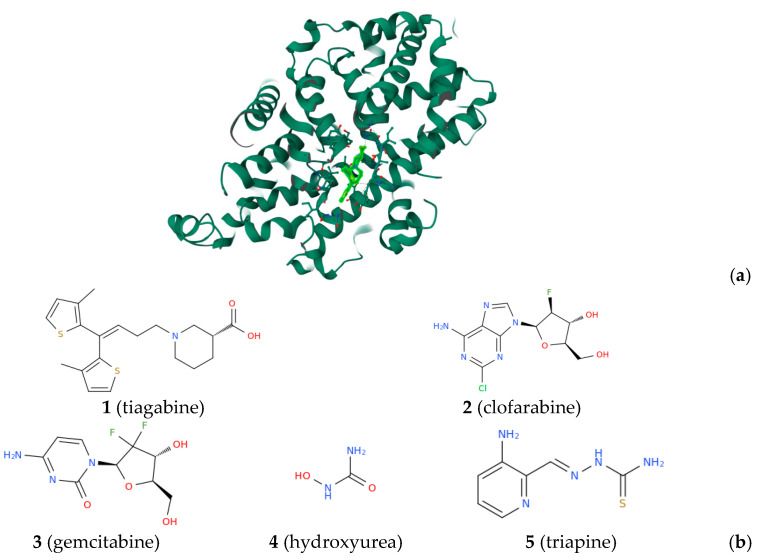
(**a**) Three-dimensional view of the GABA reuptake transporter 1 (also known as GAT-1) in complex with tiagabine (**1**) (the structure can be freely visualized at https://www.rcsb.org/3d-view/7SK2/1, accessed on 25 October 2023). (**b**) Structural representation of tiagabine, ((3R)-1-[4,4-bis(3-methylthiophen-2-yl)but-3-enyl]piperidine-3-carboxylic acid, **1**), clofarabine ((2R,3R,4S,5R)-5-(6-amino-2-chloropurin-9-yl)-4-fluoro-2-(hydroxymethyl)oxolan-3-ol, **2**), gemcitabine (4-amino-1-[(2R,4R,5R)-3,3-difluoro-4-hydroxy-5-(hydroxymethyl)oxolan-2-yl]pyrimidin-2-one, **3**), hydroxyurea (**4**), and triapine ([(E)-(3-aminopyridin-2-yl)methylideneamino]thiourea, **5**).

**Figure 2:**
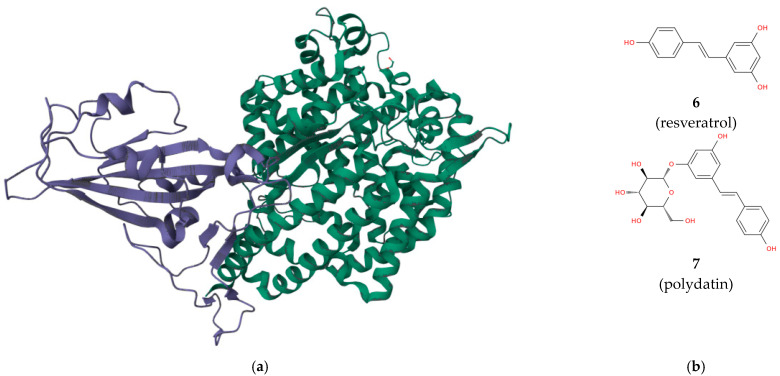
(**a**) The receptor binding domain of SARS-CoV-2 spike protein (violet) complexed with its receptor human ACE2 (green) (the structure is publicly available at https://www.rcsb.org/3d-view/6VW1/1, accessed on 25 October 2023). (**b**) Structural representation of the polyphenol resveratrol (5-[(E)-2-(4-hydroxyphenyl)ethenyl]benzene-1,3-diol, **6**) and its glycosylated form polydatin ((2S,3R,4S,5S,6R)-2-[3-hydroxy-5-[(E)-2-(4-hydroxyphenyl)ethenyl]phenoxy]-6-(hydroxymethyl)oxane-3,4,5-triol, **7**).

## References

[1] Hanna R., Dalvi S., Sălăgean T., Pop I.D., Bordea I.R., Benedicenti S. (2021). Understanding COVID-19 pandemic: Molecular mechanisms and potential therapeutic strategies. An evidence-based review. J. Inflamm. Res..

[2] Soudijn W., van Wijngaarden I. (2000). The GABA transporter and its inhibitors. Curr. Med. Chem..

[3] Madsen K.K., White H.S., Schousboe A. (2010). Neuronal and non-neuronal GABA transporters as targets for antiepileptic drugs. Pharmacol. Ther..

[4] Sałat K., Podkowa A., Mogilski S., Zaręba P., Kulig K., Sałat R., Malikowska N., Filipek B. (2015). The effect of GABA transporter 1 (GAT1) inhibitor, tiagabine, on scopolamine-induced memory impairments in mice. Pharmacol. Rep..

[5] O’Hara J.K., Kerwin L.J., Cobbold S.A., Tai J., Bedell T.A., Reider P.J., Llinás M. (2014). Targeting NAD+ metabolism in the human malaria parasite Plasmodium falciparum. PLoS ONE.

[6] Capasso D., Marino P., Di Gaetano S., Borbone N., Terracciano M., Trani R., Longo C., Piccialli V. (2023). Synthesis of Brominated Lactones Related to Mycalin A: Selective Antiproliferative Activity on Metastatic Melanoma Cells and Inhibition of the Cell Migration. Mar. Drugs.

[7] Palumbo R., Simonyan H., Roviello G.N. (2023). Advances in Amino Acid-Based Chemistry. Pharmaceuticals.

[8] Parisi E., Capasso D., Capobianco A., Peluso A., Di Gaetano S., Fusco S., Manfredi C., Mozzillo R., Pinto G., Centore R. (2020). Tautomeric and conformational switching in a new versatile N-rich heterocyclic ligand. Dalton Trans..

[9] Tramontano C., Martins J.P., De Stefano L., Kemell M., Correia A., Terracciano M., Borbone N., Rea I., Santos H.A. (2023). Microfluidic-Assisted Production of Gastro-Resistant Active-Targeted Diatomite Nanoparticles for the Local Release of Galunisertib in Metastatic Colorectal Cancer Cells. Adv. Healthc. Mater..

[10] Bothou C., Sharma A., Oo A., Kim B., Perge P., Igaz P., Ronchi C.L., Shapiro I., Hantel C. (2021). Novel insights into the molecular regulation of ribonucleotide reductase in adrenocortical carcinoma treatment. Cancers.

[11] Wijerathna S.R., Ahmad M.F., Xu H., Fairman J.W., Zhang A., Kaushal P.S., Wan Q., Kiser J., Dealwis C.G. (2011). Targeting the large subunit of human ribonucleotide reductase for cancer chemotherapy. Pharmaceuticals.

[12] Greene B.L., Kang G., Cui C., Bennati M., Nocera D.G., Drennan C.L., Stubbe J. (2020). Ribonucleotide reductases: Structure, chemistry, and metabolism suggest new therapeutic targets. Annu. Rev. Biochem..

[13] Gaur K., Pérez Otero S.C., Benjamín-Rivera J.A., Rodríguez I., Loza-Rosas S.A., Vázquez Salgado A.M., Akam E.A., Hernández-Matias L., Sharma R.K., Alicea N. (2021). Iron chelator transmetalative approach to inhibit human ribonucleotide reductase. JACS Au.

[14] Croushore E.E., Koppenhafer S.L., Goss K.L., Geary E.L., Gordon D.J. (2023). Activator Protein-1 (AP-1) Signaling Inhibits the Growth of Ewing Sarcoma Cells in Response to DNA Replication Stress. Cancer Res. Commun..

[15] Rudd S.G., Tsesmetzis N., Sanjiv K., Paulin C.B., Sandhow L., Kutzner J., Hed Myrberg I., Bunten S.S., Axelsson H., Zhang S.M. (2020). Ribonucleotide reductase inhibitors suppress SAMHD 1 ara-CTP ase activity enhancing cytarabine efficacy. EMBO Mol. Med..

[16] Poltronieri P., Miwa M., Masutani M. (2021). ADP-ribosylation as post-translational modification of proteins: Use of inhibitors in cancer control. Int. J. Mol. Sci..

[17] Horne J.R., Vohl M.-C. (2020). Biological plausibility for interactions between dietary fat, resveratrol, ACE2, and SARS-CoV illness severity. Am. J. Physiol.-Endocrinol. Metab..

[18] Ahmad I., Pawara R., Surana S., Patel H. (2021). The repurposed ACE2 inhibitors: SARS-CoV-2 entry blockers of COVID-19. Top. Curr. Chem..

[19] Agrawal P.K., Blunden G. (2023). Phytochemicals Against SARS-CoV-2 Infection.

[20] Wang M., Qin K., Zhai X. (2022). Combined network pharmacology, molecular docking, and experimental verification approach to investigate the potential mechanisms of polydatin against COVID-19. Nat. Prod. Commun..

[21] Zhang Y.P., Li Y., Xu G.C., Li J.Y., Luo H.Y., Li J.Y., Zhang L., Jia D.Z. (2019). Synthesis, crystal structure, DNA/bovine serum albumin binding and antitumor activity of two transition metal complexes with 4-acylpyrazolone derivative. Appl. Organomet. Chem..

[22] Van de Sande L., Cosyns S., Willaert W., Ceelen W. (2020). Albumin-based cancer therapeutics for intraperitoneal drug delivery: A review. Drug Deliv..

[23] Greco F., Falanga A.P., Terracciano M., D’Ambrosio C., Piccialli G., Oliviero G., Roviello G.N., Borbone N. (2022). CD, UV, and In Silico Insights on the Effect of 1, 3-Bis (1′-uracilyl)-2-propanone on Serum Albumin Structure. Biomolecules.

[24] Scognamiglio P.L., Riccardi C., Palumbo R., Gale T.F., Musumeci D., Roviello G.N. (2023). Self-assembly of thyminyl l-tryptophanamide (TrpT) building blocks for the potential development of drug delivery nanosystems. J. Nanostruct. Chem..

[25] Scognamiglio P.L., Vicidomini C., Fontanella F., De Stefano C., Palumbo R., Roviello G.N. (2022). Protein Binding of Benzofuran Derivatives: A CD Spectroscopic and In Silico Comparative Study of the Effects of 4-Nitrophenyl Functionalized Benzofurans and Benzodifurans on BSA Protein Structure. Biomolecules.

[26] Cassetta L., Pollard J.W. (2020). Tumor-associated macrophages. Curr. Biol..

[27] Liu H., He R., Yang X., Huang B., Liu H. (2023). Mechanism of TCF21 Downregulation Leading to Immunosuppression of Tumor-Associated Macrophages in Non-Small Cell Lung Cancer. Pharmaceutics.

[28] Shen C.-K., Huang B.-R., Charoensaensuk V., Yang L.-Y., Tsai C.-F., Liu Y.-S., Lu D.-Y., Yeh W.-L., Lin C. (2023). Bradykinin B1 Receptor Affects Tumor-Associated Macrophage Activity and Glioblastoma Progression. Antioxidants.

